# Nitrosative and Oxidative Stress, Reduced Antioxidant Capacity, and Fiber Type Switch in Iron-Deficient COPD Patients: Analysis of Muscle and Systemic Compartments

**DOI:** 10.3390/nu15061454

**Published:** 2023-03-17

**Authors:** Maria Pérez-Peiró, Mariela Alvarado Miranda, Clara Martín-Ontiyuelo, Diego A. Rodríguez-Chiaradía, Esther Barreiro

**Affiliations:** 1 Muscle Wasting and Cachexia in Chronic Respiratory Diseases and Lung Cancer Research Group, Pulmonology Department, Department of Medicine and Life Sciences (MELIS), Hospital del Mar, Medical Research Institute (IMIM), Parc de Salut Mar, Universitat Pompeu Fabra (UPF), Barcelona Biomedical Research Park (PRBB), 08003 Barcelona, Spain; 2 Centro de Investigación en Red de Enfermedades Respiratorias (CIBERES), Instituto de Salud Carlos III (ISCIII), 08003 Barcelona, Spain; 3 Department of Pulmonary Medicine, Hospital Universitari Sant Joan, 43204 Reus, Tarragona, Spain; 4 Department of Pulmonary Medicine, Hospital Clínic-Institut d’Investigacions Biomèdiques August Pi i Sunyer (IDIBAPS), University of Barcelona, 08036 Barcelona, Spain

**Keywords:** COPD, non-anemic iron deficiency, nitrosative stress, antioxidant systems, lipofuscin inclusions, muscle fiber type switch, muscle and systemic compartments

## Abstract

We hypothesized that a rise in the levels of oxidative/nitrosative stress markers and a decline in antioxidants might take place in systemic and muscle compartments of chronic obstructive pulmonary disease (COPD) patients with non-anemic iron deficiency. In COPD patients with/without iron depletion (*n* = 20/group), markers of oxidative/nitrosative stress and antioxidants were determined in blood and vastus lateralis (biopsies, muscle fiber phenotype). Iron metabolism, exercise, and limb muscle strength were assessed in all patients. In iron-deficient COPD compared to non-iron deficient patients, oxidative (lipofuscin) and nitrosative stress levels were greater in muscle and blood compartments and proportions of fast-twitch fibers, whereas levels of mitochondrial superoxide dismutase (SOD) and Trolox equivalent antioxidant capacity (TEAC) decreased. In severe COPD, nitrosative stress and reduced antioxidant capacity were demonstrated in vastus lateralis and systemic compartments of iron-deficient patients. The slow- to fast-twitch muscle fiber switch towards a less resistant phenotype was significantly more prominent in muscles of these patients. Iron deficiency is associated with a specific pattern of nitrosative and oxidative stress and reduced antioxidant capacity in severe COPD irrespective of quadriceps muscle function. In clinical settings, parameters of iron metabolism and content should be routinely quantify given its implications in redox balance and exercise tolerance.

## 1. Introduction

Patients with chronic respiratory and cardiac disorders, such as chronic obstructive pulmonary disease (COPD), experience systemic manifestations and comorbidities that affect different organs other than the lungs and airways, particularly in patients with a more advanced disease [1,2]. Skeletal muscle weakness and nutritional abnormalities are encountered among the most prominent manifestations in COPD due to their clinical implications in disease prognosis, including overall survival and impaired quality of life [1,2,3].

The transition metal iron (Fe) is involved in several key cellular processes in mammals. In humans, iron is mostly found in hemoglobin and myoglobin proteins that are responsible for oxygen transport in blood and oxygen storage in muscles, respectively. Furthermore, iron is also present in the active site of redox enzymes that are involved in important chemical reactions, such as respiration, oxidation, and reduction within the cells [4,5,6]. A minimum iron uptake from the diet is necessary in order to keep the required homeostatic levels in humans [4,5,6].

However, in the elderly and in patients with chronic heart and renal failure, the levels of iron may be severely lowered [7,8,9]. In patients with COPD, iron depletion has also been documented in previous investigations [10,11,12,13,14]. In a recent study [15], a potential crosstalk between systemic and muscle compartments was observed in COPD patients with iron deficiency. Moreover, lung function and exercise capacity were associated with several markers involved in the regulation of iron metabolism in the same cohort of patients [15]. Hence, in iron content depletion is common among patients with chronic diseases, including COPD [10,11,12,16,17].

Oxidative stress defined as the imbalance between prooxidants and antioxidants in favor of the former has been shown to induce damage to cells and tissues. Increased levels of oxidative and nitrosative stress have been consistently demonstrated in the vastus lateralis muscle of patients with COPD [18,19,20,21]. Enhanced proteolysis of major structural proteins is a relevant deleterious effect of oxidative stress within the myofibers, leading to muscle atrophy and even sarcopenia in patients with COPD [18,19,20,21,22,23]. Other mechanisms, such as apoptosis and autophagy, may also be signaled by high levels of reactive oxygen species (ROS) within the skeletal muscle fibers [21,24,25,26]. ROS are generated by different sources in the cells and skeletal muscle fibers. Moreover, Fenton reactions between iron and hydrogen peroxide also lead to the formation of the powerful ROS hydroxyl radicals, with a great potential to oxidize other molecules in cells [27]. Whether variations in the levels of iron content may also alter the levels of oxidative stress in the muscle fibers of patients with COPD without anemia warrants further attention. The analysis of whether changes in iron levels may also influence the muscle phenotype in COPD also remains to be answered.

Iron deficiency anemia has been reliably associated with a rise in oxidative stress levels in the systemic compartment and the myocardium [28,29,30,31,32]. The levels of antioxidants were also significantly reduced in patients with iron-deficiency anemia [28,32]. Whether iron depletion without anemia may also entail an increase in oxidative stress levels in patients with COPD needs to be thoroughly investigated. On this basis, we hypothesized that a rise in the levels of oxidative/nitrosative stress markers along with a decline in antioxidants may take place in the systemic and muscle compartments of COPD patients with non-anemic iron deficiency. The study objectives were that in non-anemic COPD patients with and without iron depletion, markers of oxidative and nitrosative stress and antioxidants were determined in blood and vastus lateralis muscle specimens along with characterization of the muscle fiber phenotype. Additionally, all the patients were clinically evaluated, including iron metabolism parameters, and both exercise capacity and limb muscle strength were also determined. For the purpose of the investigation, two different experimental groups were recruited: COPD patients with non-anemic iron deficiency and COPD patients with normal iron content as the control group.

## 2. Materials and Methods

### 2.1. Study Population

This was a cross-sectional study, in which forty severe COPD patients were recruited. The Global Strategy of Management of COPD patients (GOLD) was used to diagnose and classify the patients according to disease severity [33]. Clinical parameters and dyspnea score (modified medical research council, mMRC) were obtained from all the participants [34]. All patients were recruited during the years 2018–2021 in the Pulmonology Department at Hospital del Mar (Barcelona, Spain). The following criteria were used to define iron deficiency: hemoglobin > 12 g/dL in women and >13 g/dL in men, ferritin < 100 ng/mL, or ferritin 100–299 ng/mL with a transferrin saturation <20% [16,35]. Iron deficiency without anemia was detected in half of the patients. Hence, on the basis of the iron status, patients were subdivided into two different groups: iron deficiency and non-iron deficiency patients (N = 20/group, respectively, 12 male patients/group).

The current investigation was approved by the Ethics Committee on Human Investigation at Hospital de Mar (Hospital del Mar-IMIM, Barcelona, project number # 2017/7691/I). Ethical standards on human experimentation from our institution, the World Medical Association guidelines (Seventh revision of the Declaration of Helsinki, Fortaleza, Brazil, 2013) [36], and the guidelines established by the International Committee of Medical Journal Editors (ICMJE) were followed. All the participants signed the informed written consent.

### 2.2. Exclusion Criteria

The following exclusion criteria were established: (1) acute exacerbations in the last three months; (2) other chronic diseases with lung and airways implications (e.g., long-term oxygen therapy, bronchiectasis, asthma); (3) cardiovascular disorders; (4) musculoskeletal alterations; (5) neurological, metabolic, kidney, chronic liver disease, or uncontrolled psychiatric disorders; (6) obesity (body mass index > 30 Kg/m^2^); (7) pharmacological treatment with drugs known to alter muscle structure and/or function including oral corticosteroids; (8) active oncologic disease; and (9) history of potentially bleeding conditions.

### 2.3. Clinical Assessment

In all the patients, lung function was measured through conventional spirometry and references values for a Mediterranean population were taken [37,38,39]. Anthropometric evaluation included body mass index (BMI) and fat-free mass index (FFMI), which was measured using bioelectrical impedance in all the participants [40,41]. The six-minute walk test was used to determine the exercise capacity in all the patients [42]. The six-minute walk test was conducted along a 30-m indoor flat corridor with no obstacles. Patients were encouraged every minute and were allowed to rest if needed to resume the exercise as soon as they could walk again. The test lasted for six minutes in all the patients independently of whether they had to stop to rest.

Upper (handgrip) and lower (quadriceps) limb muscle function was evaluated in all the participants. As such, the maximum voluntary contraction of the flexor muscles was measured using a specific hand dynamometer (Jamar 030J1, Chicago, IL, USA). Reference values from Luna-Heredia et al. [43] were used. Quadriceps muscle strength was quantified through the determination of the isometric maximum voluntary contraction (QMVC) following standard procedures [44]. Briefly, a fixed handheld dynamometer [MicroFet 2^TM^, Hoggan Scientific, Salt Lake City, UT, USA] was placed on the tibia and the QMVC was obtained through the exerted compression force. In both tests, three reproducible measurements (<5% variability among them) were obtained in each patient. The highest value out of three maneuvers was selected as the handgrip or QMVC measurements. Reference values from Seymour et al. [45] were used.

### 2.4. Blood Samples

After an overnight fasting period, blood samples were drawn from the arm vein of each patient. Serum specimens were obtained through the centrifugation of blood samples collected into Vacuette^®^ serum tubes (with clot activator) at 1600× *g* for 15 min. Serum samples were immediately stored in the −80 °C freezers until further use.

### 2.5. Muscle Biopsies

As previously described, specimens from the vastus lateralis were obtained using the open biopsy technique from all the study patients [15,19,20,21,23]. Muscle samples were immediately frozen in liquid nitrogen and then stored at −80 °C (temperature under alarm control) for the molecular biology experiments. The second half of the muscle specimen was immersed in a series of alcohol baths to be thereafter embedded in paraffin. Paraffin-embedded samples were used for the purpose of the structural analyses carried out on the muscle specimens from all the patients.

### 2.6. Biological Analyses

*Systemic iron status.* Conventional analytical parameters were analyzed in the systemic compartment in all the patients: ferritin, transferrin saturation total iron, transferrin, soluble transferrin receptor, hepcidin, hematocrit, hemoglobin, mean corpuscular (erythrocyte) volume (MCV), mean corpuscular hemoglobin (MCH), and mean corpuscular hemoglobin concentration (MCHC).

*Hepcidin-25.* Levels of hepcidin-25 concentration were quantified in serum samples using the Human hepcidin (Hepc) ELISA kit (Biorbyt, Cambridgeshire, UK) following the manufacturer’s instructions and previously described methodologies [14]. In each well of the hepcidin antibody pre-coated microplate, 50 µL 5-fold diluted serum samples or standard and 50 µL HRP-conjugate were poured. Samples were then incubated at 37 °C for 1 h and were washed three times. Moreover, 50 µL of substrate A and 50 µL of substrate B were added and incubated at 37 °C for 15 min. The enzymatic reaction was stopped by adding 50 µL stop solution. Absorbance of each sample was read at a 450 nm wavelength in a plate-reader (Infinite M Nano, Tecan Group Ltd., Zürich, Switzerland). A standard curve was always generated with each assay run. Intra-assay coefficients of variation (CV) for all the samples ranged from 0.1% to 11.0%. The intra-assay CV for all the analyzed markers was calculated as a result of dividing the standard deviation of each replicate value by its mean, which was then multiplied by 100. Standard curves data and intra-assay CVs of all kits used in this study are shown in the Supplementary Materials.

*Protein tyrosine nitration*. Levels of nitrated proteins were analyzed using the Elabscience^®^ 3-NT(3-Nytrotyrosine) ELISA kit (Elabscience, Houston, TX, USA) in the serum samples of all the patients following standard procedures and previous investigations [14,46]. All reagents were equilibrated at room temperature before the beginning of the ELISA procedures. Thus, 50 µL 7-fold diluted serum samples and standards were added in the corresponding wells of the 3-nitrotyrosine antibody pre-coated microplates. Samples were incubated at 37 °C with the biotinylated antibody for 45 min. After three consecutive washes, HRP conjugate working solution was added to each well, and samples were then incubated at 37 °C for 30 min. Finally, after five additional washes, samples were incubated at 37 °C with the substrate reagent for 15 more minutes. Following this incubation, enzyme substrate reaction was stopped by adding the stop solution. Optical densities in each well were determined by reading the absorbance of the samples at a 450 nm wavelength in a plate-reader (Infinite M Nano, Tecan Group Ltd., Zürich, Switzerland). A standard curve was always generated with each assay run. Intra-assay coefficients of variation for all the samples ranged from 0.27% to 9.81%.

*Reactive carbonyls in proteins.* The Protein Carbonyl Content Assay Kit (Sigma-Aldrich, St. Louis, MO, USA) was used to assess the levels of reactive carbonyls in serum samples of the study patients following standard procedures published in previous reports [47,48]. The protein concentrations of serum samples in all study patients were determined using the Pierce BCA Protein Assay Kit (Thermo Scientific, Rockford, IL, USA). Samples were diluted with purified water to a protein concentration of 5 mg/mL. All samples and standards were run in duplicate. Samples were incubated at room temperature for 15 min with 10 μL of the 10% Streptozocin solution per 100 μL of sample to degrade the remaining nucleic acids of samples. Following this incubation, samples were centrifuged at 13,000× *g* for 5 min and then the supernatant was transferred to a new tube. Afterwards, 100 μL of DNPH solution was added to each sample and gently vortexed. After an incubation of 10 min at room temperature, 30 μL of the 87% TCA solution was poured to each sample and gently vortexed. Samples were incubated with TCA solution on ice for 5 min and then centrifugated at 13,000× *g* for 2 min. Supernatant was careful removed to not disturbed the pellet and 500 μL of ice-cold acetone was added to each pellet and placed in a sonication bath for 30 s. Subsequently, samples were incubated at −20 °C for 5 min and then centrifuged at 13,000× *g* for 2 min. Acetone were carefully removed from pellet and then 200 μL of 6 M Guanidine solution was poured to pellet and sonicate briefly. Successively, pellets were spined briefly to remove any insolubilized material. Then, 100 μL of each sample was transferred to the 96-well plate to analyze the levels of serum protein carbonylation and 5 μL of the remaining sample was used to determine the protein concentration of each sample. Absorbances were read in each well at a 450 nm wavelength in a plate-reader (Infinite M Nano, Tecan Group Ltd., Zürich, Switzerland) to detect serum levels of protein carbonyls. A standard curve was always generated with each assay run. Intra-assay coefficients of variation for all the samples ranged from 0.24% to 6.43%.

*Levels of malondialdehyde-protein adducts.* The OxiSelect^TM^ MDA Adduct Competitive ELISA Kit (Cell Biolabs, Inc., San Diego, CA, USA) was used to quantify the levels of MDA-protein adducts in serum samples of the study patients following standard procedures as previously reported [14,49]. Fifty µL serum and MDA-BSA standards were mixed with the MDA conjugate preabsorbed ELISA plate. Samples were then incubated at room temperature for 10 min. Subsequently, the primary antibody was added and incubated at room temperature for one hour. After three washes, samples were incubated with the HRP conjugated secondary antibody at room temperature for an hour. Subsequently, the plate was washed three more times and the substrate solution was incubated at room temperature for 20 min. The enzyme reaction was stopped by adding 100 µL stop solution into each well. Absorbances were read in each well at a 450 nm wavelength in a plate-reader (Infinite M Nano, Tecan Group Ltd., Zürich, Switzerland). A standard curve was always generated with each assay run. Intra-assay coefficients of variation for all the samples ranged from 0.13% to 9.74%.

Activity of superoxide Dismutase (SOD). The Superoxide Dismutase Assay Kit (Cayman chemical, Ann Arbor, MI, USA) was used to quantify the levels of SOD activity in serum samples of all the patients following standard procedures that were reported previously [14,21,49]. Reagents were equilibrated to room temperature before beginning the assay. Serum samples were diluted 1:5 with sample buffer before assaying for SOD activity. Subsequently, 10 µL diluted samples, standards, and 200 µL of the diluted Radical Detector were added in the designated wells on the plates. To initiate the enzymatic reaction, 20 µL xanthine oxidase was added to the wells. Samples were incubated at room temperature for 30 min and the absorbances were read at 440 nm in a plate-reader (Infinite M Nano, Tecan Group Ltd., Zürich, Switzerland). A standard curve was always generated with each assay run. Intra-assay coefficients of variation for all the samples ranged from 0.12% to 8.92%.

*Catalase activity*. The Catalase Assay Kit (Cayman chemical, Ann Arbor, MN, USA) was used to analyze catalase activity in the serum samples of all the patients following standard procedures that had been previously reported [14,21,49]. All reagents were equilibrated to room temperature before beginning the assay. Briefly, 20 µL samples, standards, and positive control were diluted in 100 µL assay buffer, which were added to the corresponding wells. Subsequently, 30 µL methanol was poured into each well and the reaction took place by adding 20 µL hydrogen peroxide. Following a 20-minute incubation on a shaker at room temperature, 30 µL potassium hydroxide was added to terminate the reaction. Subsequently, 30 µL catalase purpald (chromogen) was incubated with the samples for ten minutes. Finally, 10 µL catalase potassium periodate was incubated with the samples at room temperature for 5 min and the absorbances were read at a 540 nm wavelength in a plate-reader (Infinite M Nano, Tecan Group Ltd., Zürich, Switzerland). A standard curve was always generated with each assay run. Intra-assay coefficients of variation for all the samples ranged from 0.42% to 9.25%.

*Levels of reduced glutathione (GSH)*. The Human Reduced Glutathione (GSH) ELISA Kit (MyBioSource, San Diego, CA, USA) was used to determine GSH levels in serum samples of all the patients following standard procedures that were previously reported [14,46]. All reagents and samples needed to be equilibrated to room temperature (18–25 °C) before starting the procedure. Briefly, 50 µL samples and standards were added to each well and incubated with horseradish (HRP)-conjugate reagent at 37 °C for 60 min. After four washes, 50 µL of chromogen solution A and 50 µL of chromogen solution B were added to each well. Samples were then incubated at 37 °C in the dark for 15 min. Finally, 50 µL of the stop solution was incubated for five minutes, and the absorbance in each sample was read at a 450 nm wavelength in a plate-reader (Infinite M Nano, Tecan Group Ltd., Zürich, Switzerland). A standard curve was always generated with each assay run. Intra-assay coefficients for all the samples ranged from 0.06% to 7.78%.

*Levels of Trolox Equivalent Antioxidant Capacity (TEAC)*. The OxiSelectTM TEAC (TEAC Assay Kit (ABTS) (Cell Biolabs, Inc., San Diego, CA, USA) was used to analyze the total antioxidant capacity of the serum samples in all the patients following standard procedures previously reported [14,50]. Briefly, 25 µL of 20-fold diluted serum samples were added to each well. Subsequently, 150 µL of the diluted 2,2′-azino-bis (3-ethylbenzothiazoline-6-sulfonic acid) (ABTS) reagent was mixed vigorously with the samples to be incubated for five minutes. Finally, the absorbances were read at a 405 nm wavelength in a plate-reader (Infinite M Nano, Tecan Group Ltd., Zürich, Switzerland). A standard curve was always generated with each assay run. Antioxidant activity was determined by comparison with the Trolox standards. Intra-assay coefficients of variation for all the samples ranged from 0.07% to 5.28%.

### 2.7. Muscle Redox Markers

*Tissue homogenization and protein quantification.* Protein homogenates were obtained from all the frozen muscle specimens following standard procedures [21,51]. Briefly, frozen specimens of vastus lateralis were mechanically homogenized in specific lysis buffer containing 50 mM 4-(2-hydroxyethyl)-1-piperazineethanesulfonic acid (HEPES), 150 mM NaCl, 100 nM NaF, 10 mM Na pyrophosphate, 5 mM EDTA, 0.5%Triton-X, 2 μg/mL leupeptin, 100 μg/mL phenylmethylsulfonyl fluoride (PMSF), 2 μg/mL aprotinin, and 10 μg/mL pepstatin A using a tissue homogenizer. Samples were kept on ice during the entire procedure. After homogenization, samples were centrifugated at 3600 rpm, at 4 °C for 30 min. Then, pellets were discarded, and supernatants were aliquoted to further protein quantification. The Bradford methodologies were used to analyze protein concentration [21,51].

*Immunoblotting procedures.* Protein samples (5–20 µg, according to antigen and antibody) were diluted 1:1 with 2X Laemmli buffer (Bio-Rad Laboratories, Inc., Hercules, CA, USA) and 10% of 2-mercaptoethanol (Bio-Rad Laboratories, Inc., Hercules, CA, USA). The samples were then boiled at 95 °C for five minutes to be exposed to electrophoretic separation according to their molecular weight. After the electrophoresis, the proteins were transferred onto polyvinylidene difluoride (PVDF) membranes (Merck KGaA, Darmstadt, Germany) to be blocked with bovine serum albumin (BSA) (NZYTech, Lisbon, Portugal) or 5% nonfat milk, depending on the antibody. Immediately afterwards, the membranes were incubated with the corresponding primary antibodies at 4 °C (cold room) overnight. The following antibodies were used in order to determine the target biomarkers in the study: reactive carbonyls in proteins (protein carbonyl assay kit, Abcam, Cambridge, UK), MDA-protein adducts (anti-MDA-protein adducts antibody, Academy Bio-Chemical Company, Houston, TX, USA), protein tyrosine nitration as measured by levels of 3-nitrotyrosine (anti-3-nitrotyrosine antibody, Thermo Fisher Scientific, Waltham, MA, USA), SOD-1 (anti-SOD-1 antibody, Santa Cruz Biotechnology, Dallas, TX, USA), SOD-2 (anti-SOD-2 antibody, Santa Cruz Biotechnology), catalase (anti-catalase antibody, Merck KGaA, Darmstadt, Germany), and the loading control glyceraldehyde-3-phosphate dehydrogenase (GAPDH, anti-GAPDH antibody, Santa Cruz Biotechnology). The corresponding antigens were detected using HRP-conjugated secondary antibodies (Jackson ImmunoResearch Inc., West Grove, PA, USA) and a chemiluminescence kit (Thermo Scientific, Rockford, IL, USA). The Alliance Q9 Advanced (Uvitec, Cambridge, UK) equipment was used to identify each of the antigens in the PVDF membranes. The forty samples analyzed in the study were always exposed to identical conditions during the entire immunoblotting procedures, including chemiluminescence detection and image capturing. The optical densities of the specific protein bands were quantified using the ImageJ software (National Institute of Health, available at http://rsb.info.nih.gov/ij/, accessed on 1 November 2022). The optical densities were normalized with those of the glycolytic enzyme GAPDH in all the immunoblots. Negative control experiments in which primary antibodies were omitted were also performed in the study. For technical-methodological reasons, nineteen and twenty muscle specimens were analyzed in COPD patients without and with iron deficiency, respectively.

### 2.8. Muscle Phenotype and Damage

*Muscle fiber composition and morphometry.* The proteins MyHC-I and -II isoforms were identified to analyze muscle fiber phenotype using immunohistochemistry [40,41,51]. Following a deparaffination process with xylene and a graded ethanol series (rehydration), samples were subject to antigen retrieval. As such, samples were incubated with 1 mM EDTA buffer with 0.05% Tween20 (pH 8.0) at 95 °C for forty minutes, to be cooled down for another thirty minutes. Endogenous peroxidase activity was blocked with 6% H_2_O_2_. Incubation with the corresponding primary antibodies, anti-MyHCI and anti-MyHCII antibodies (Abcam, Cambridge, UK), respectively, at room temperature for 40 min followed. Sections were than washed three times with PBS. Subsequently, afterwards, slides were incubated with horseradish peroxidase (HRP) Polymer-antiMouse/Rabbit IgG at room temperature for thirty minutes (Neobiotech, Seoul, Republic of Korea). After three more washes with PBS, slides were then incubated with 3,3′-Diaminobenzidine (DAB) (Neobiotech, Seoul, Republic of Korea) solution until the appropriate color (brown) was reached. Subsequently, sections were rinsed with tap water to be counterstained with hematoxylin. Finally, all the muscle sections were dehydrated in a battery of alcohol and xylene solutions to be mounted in dibutylphthalate polystyrene xylene (DPX) media (Sigma-Aldrich, St. Louis, MO, USA). A negative control section (primary antibody omission) was always prepared in each sample. Images were captured using a light microscope (x20 objective, Olympus, Series BX50F3, Olympus Optical Co., Hamburg, Germany) to be analyzed. In all the muscle sections, the following items were assessed: cross-sectional area, mean least diameter, and the proportions of type I, type II, and those of the hybrid muscle fibers. The morphometric analysis was performed using ImageJ software (National Institute of Health, available at http://rsb.info.nih.gov/ij/, accessed on 1 November 2022). In each muscle cross-section, at least 100 fibers were measured in both groups of patients. The proportions of fiber types were also counted in each muscle preparation from both groups of patients. For technical-methodological reasons, fiber morphometry and composition analyses were assessed in eighteen and sixteen muscle specimens from in COPD patients without and with iron deficiency, respectively.

*Muscle structural abnormalities.* Structural abnormalities were assessed on the 3-μm paraffin-embedded muscle sections following standard procedures [51,52]. Images of the stained sections were captured using a light microscope (×40 objective, Olympus, Series BX50F3, Olympus Optical Co., Hamburg, Germany). The images were superimposed on a 63-square grid composed of a 7 × 9 rectangular pattern to analyze the abnormal and normal muscle fractions through the evaluation of several markers: (1) normal muscle, (2) internal nucleus, (3) inflammatory cell, (4) lipofuscin, (5) abnormal viable fiber, (6) inflamed/necrotic fiber, (7) blood vessel, and (0) no count. The normal muscle fraction was calculated as the percentage of all the points that fell into the first category relative to the total number of points counted on the viable fields, whereas the abnormal fraction was defined as the percentage of points that fell between categories 2 and 6 relative to the total number of counted points. Categories 0 and 7 were not considered in the counting.

*Terminal deoxynucleotidyl transferase-mediated dUTP nick-end labeling (TUNEL) assay*. The TUNEL assay (ApopTag Peroxidase In Situ Apoptosis Detection Kit; Merck Millipore) was used to identify the apoptotic nuclei [53,54]. In brief, DNA strand breaks that are generated during nuclear activation can be identified by labeling the 3′-OH terminal groups. The labeling of the 3′-OH groups with modified nucleotides is carried out by an enzymatic reaction catalyzed by the terminal deoxynucleotidyl transferase (TdT) enzyme. Muscle sections were fixed, permeabilized, and immediately incubated with the TUNEL Working Strength TdT Enzyme and the anti-Digoxigenin Conjugate. TdT catalyzed the adding of digoxigenin-dNTP at 3′-OH terminal groups in single- and double-stranded DNA. After several washes, the digoxigenin-nucleotide that were bound to DNA fragments were detected using an anti-digoxigenin antibody conjugated with peroxidase, which resulted in a brown color upon reaction. Methyl green counterstaining was additionally performed to distinguish the negatively stained nuclei. Negative control experiments, in which the TdT enzyme was not added, were also performed. Only the nuclei located within the muscle fiber boundary were counted in the study. The final count of positive and total nuclei was carried out by two trained observers (correlation coefficient 95%). Apoptotic nuclei were expressed as the percentage of the TUNEL-positive nuclei to the total number of counted nuclei in each muscle preparation [53,54].

### 2.9. Statistical Analysis

The normality of all variables was analyzed using the Shapiro –Wilk test and histograms. The normality of the study variables determined the test used to assess potential differences between the two groups: the independent-sample Student’s *t* test (parametric) and the Mann–Whitney U test (nonparametric). The Chi-squared test was used to analyze differences for the categorical variables between the two groups. Potential associations were assessed using the Pearson’s correlation coefficient. Such correlations were explored within each group of patients individually and as whole. Clinical characteristics of the patients are presented in tables, while results from the biological analyses are depicted in graphs. The results are expressed as mean and standard deviation for each variable in each study group. In the tables, results are presented as mean and standard deviation in numbers, while individual data points are shown in the graphs. Moreover, the fiber type distributions in both patient groups are represented in histograms of the percentages of slow- and fast-twitch muscle fibers in a specific figure.

For the calculation of the sample size, the hepcidin was used as the target variable. In a two-sided test, to accept an α-risk of 0.05 and a β-risk of 0.2 (80% power), at least 16 patients in each group were required to detect a minimum difference of 200 ng/mL in hepcidin levels between the two groups. Statistical significance was established at *p* ≤ 0.05. All the statistical analyses were performed using the software SPSS 23.0 (SPSS Inc., Chicago, IL, USA).

## 3. Results

### 3.1. Clinical Characteristics of the Study Patients

Anthropometry, smoking history, lung function, and GOLD classification were similar in both groups of patients (Table 1). In iron-deficiency patients, serum levels of ferritin, transferrin saturation, and hepcidin were lower, while those of soluble transferrin receptor and transferrin were higher than in the non-iron deficiency patients (Table 1). The six-minute walk test distance was reduced (absolute and reference values) in iron-deficiency COPD patients (Table 2). Muscle strength of the upper and lower limb muscles did not significantly differ between the two study groups (Table 2).

### 3.2. Pro-Oxidant Markers in Vastus Lateralis and Blood of COPD Patients

**Figure 1:**
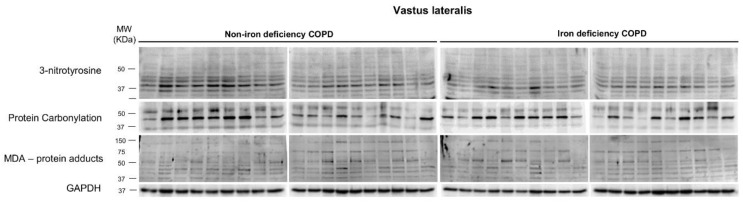
Representative immunoblots of 3-nitrotyrosine, protein carbonylation, MDA-protein adducts, and GAPDH proteins in the vastus lateralis of all COPD patients. Definition of abbreviations: MDA, malonaldehyde; GAPDH, glyceraldehyde-3-phosphate dehydrogenase; MW, molecular weight; kDa, kilodalton.

**Figure 2:**
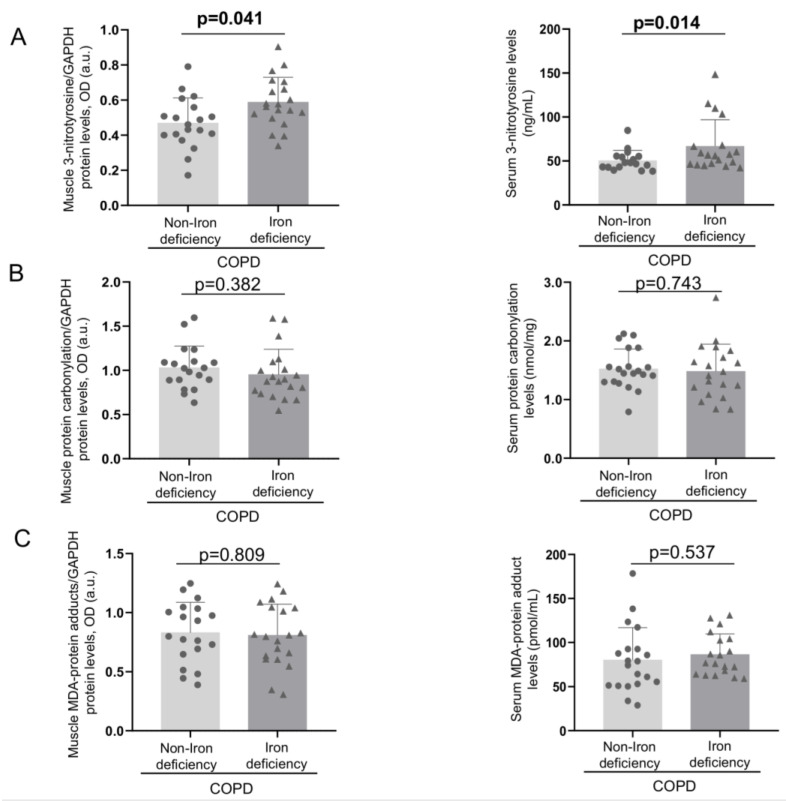
Mean values and standard deviation of 3-nitrotyrosine (**A**), protein carbonylation (**B**), and MDA-protein adducts levels (**C**) in vastus lateralis (left-hand side panels, N = 19 non-iron deficient COPD, N = 20 iron-deficient COPD) and serum (right-hand side panels, N = 20 in each group). Definition of abbreviations: MDA, malonaldehyde; GAPDH, glyceraldehyde-3-phosphate dehydrogenase; MW, molecular weight; kDa, kilodalton. Muscle protein content was measured by optical densities in arbitrary units (OD, a.u.).

Protein tyrosine nitration levels were greater in both muscle and blood compartments of iron-deficiency than in the non-iron deficiency COPD patients (Figure 1 and Figure 2A). No significant differences were seen in either protein carbonylation or MDA-protein adducts in any of the compartments between the two patient groups (Figure 1 and Figure 2B,C).

### 3.3. Antioxidants Markers in Vastus Lateralis and Blood of COPD Patients

**Figure 3:**
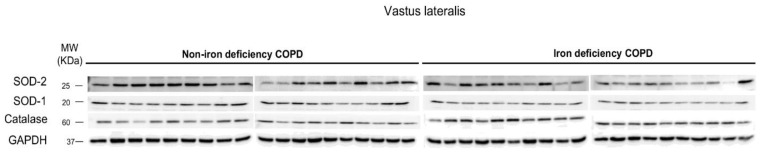
Representative immunoblots of SOD-2, SOD-1, catalase, and GAPDH proteins in the vastus lateralis of all COPD patients. Definition of abbreviations: SOD, superoxide dismutase; GAPDH, glyceraldehyde-3-phosphate dehydrogenase; MW, molecular weight; kDa, kilodalton.

**Figure 4:**
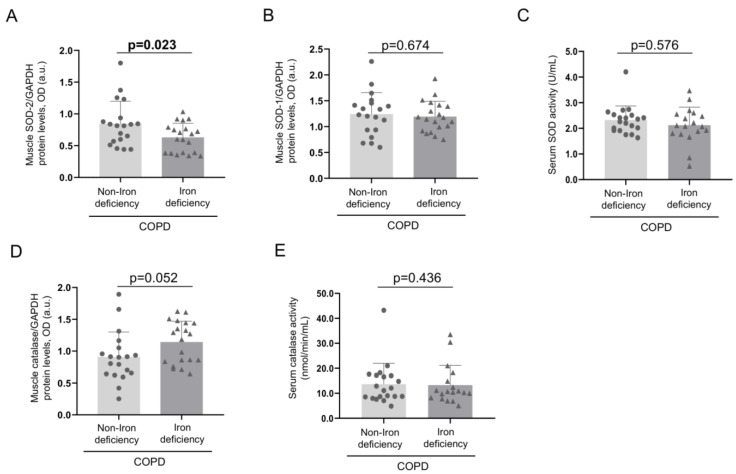
Mean values and standard deviation of muscle protein content of SOD-2 (**A**), SOD-1 (**B**), and serum SOD activity (**C**). Mean values and standard deviation of muscle protein content of catalase (**D**) and serum catalase activity (**E**). Muscle protein content was assessed in 19 non-iron deficiency and 20 iron-deficient COPD patients, while the activity of the target enzymes was analyzed in the serum of all the study patients (n = 20/group). Definition of abbreviations: SOD, superoxide dismutase; GAPDH, glyceraldehyde-3-phosphate dehydrogenase.

**Figure 5:**
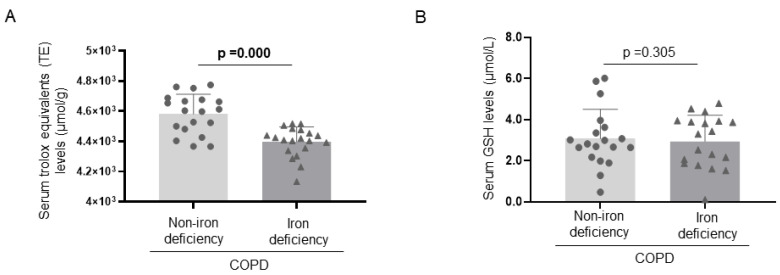
Mean values and standard deviation of serum TEAC levels (**A**) and serum GSH levels (**B**) (N = 20 in each group). Definition of abbreviations: GSH, reduced glutathione.

Protein levels of mitochondrial SOD were significantly lower in the vastus lateralis of iron-deficiency than in the non-iron deficiency patients (Figure 3 and Figure 4A). No significant differences in levels of muscle SOD-1 protein content or serum SOD activity were detected between the two study groups (Figure 3 and Figure 4B,C). Muscle protein content or serum activity levels of catalase did not significantly differ between the two COPD groups of patients (Figure 4D,E). Total antioxidant activity measured as TEAC levels significantly decreased in the iron- deficiency group compared to the non-iron deficiency patients (Figure 5A). Serum antioxidant GSH levels did not significantly differ between the two study groups (Figure 5B).

### 3.4. Muscle Structural Features of the Study Patients

**Figure 6:**
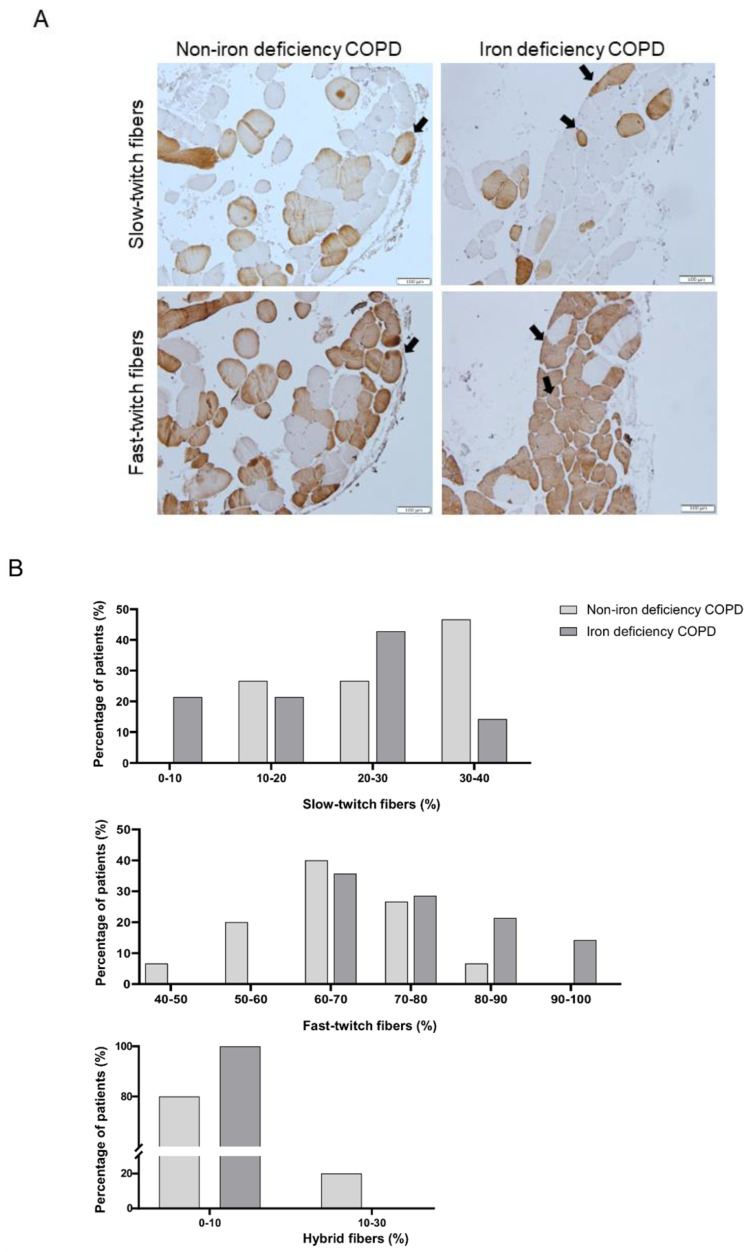
Representative images of vastus lateralis muscle fibers. Myofibers with anti-MyHC type I antibody staining (brown color) are shown in the top panel and anti-MyHC type II antibody staining in the bottom panel. Hybrid fibers (arrows) are seen in both panels. Scale bar = 100 μm, 20× magnification (**A**). Histograms of the percentages of patients (ordinate axis) of the fiber type distributions (abscissa axis by ranges) of slow-twitch (top panel), fast-twitch (middle panel), and hybrid (bottom panel) muscle fibers in the two study groups of patients (N = 18 non-iron deficient COPD, N = 16 iron-deficient COPD) (**B**).

**Figure 7:**
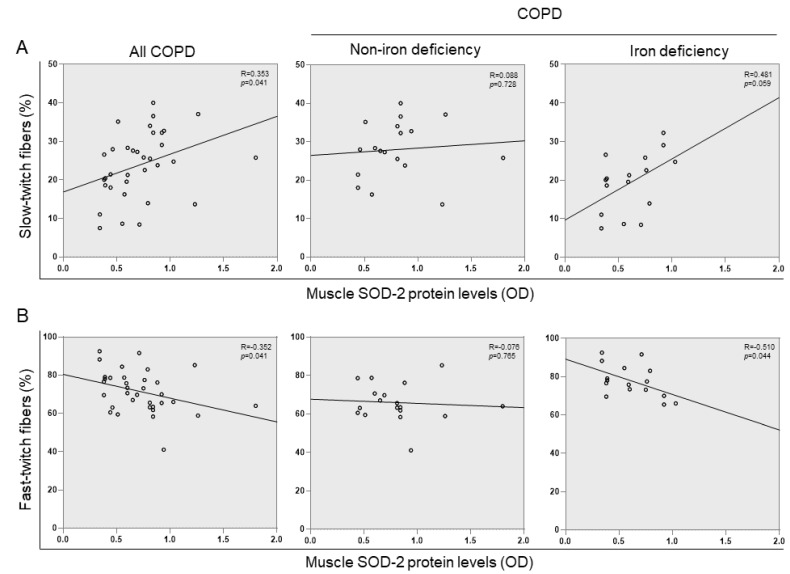
Scatter plots representation of correlations between muscle SOD-2 protein levels and the percentage of slow-twitch fibers (**A**) and fast-twitch fibers (**B**) in all COPD patients (left panels, N = 34), non-iron deficiency (middle panels, N = 18), and iron deficiency (right panels, N = 16) COPD patients. Definition of abbreviations: COPD, chronic obstructive pulmonary disease.

**Figure 8:**
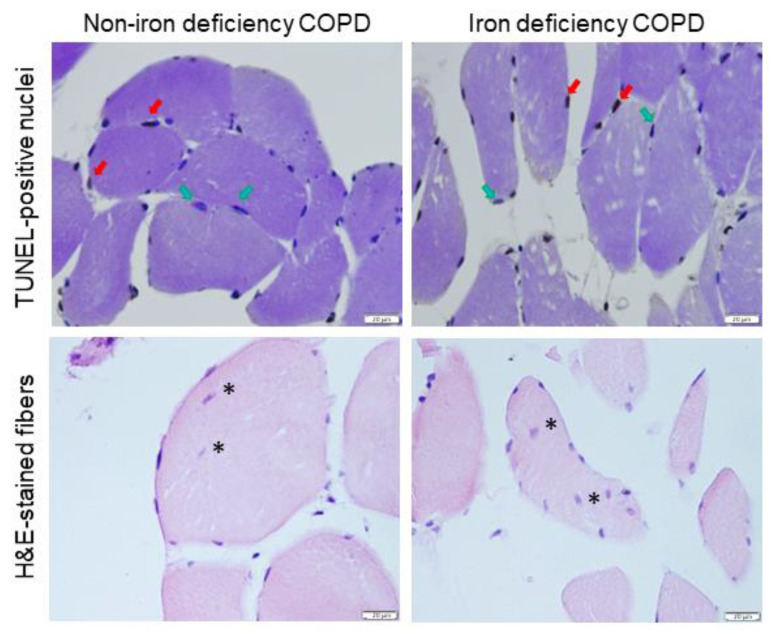
Representative images of TUNEL-positive nuclei (**top panel**) and muscle morphology within the vastus lateralis (**bottom panel**). Examples of TUNEL-positively stained nuclei (brown, red arrows), TUNEL-negative nuclei (purple, blue arrows), and internal nuclei (asterisks) are indicated in the panels. Definition of abbreviations: COPD, chronic obstructive pulmonary disease; TUNEL, Terminal deoxynucleotidyl transferase dUTP nick end labeling; H&E, hematoxylin and eosin. Scale bar = 20 μm, 40× magnification.

The proportions of fast-twitch fibers increased in the vastus lateralis of the iron-deficiency compared to the non-iron deficiency patients, whereas the percentages of the slow-twitch fibers significantly declined in the former group (Table 3, Figure 6A,B). The cross-sectional area of the analyzed muscle fibers did not significantly differ between the two study groups (Table 3, Figure 6A). The proportions or size of the hybrid fibers did not significant differ between the two study groups. The proportions of the slow-twitch fibers positively correlated with muscle SOD-2 protein content among all COPD patients (Figure 7A). This correlation was lost when non-iron deficiency COPD patients were analyzed independently, whereas in the iron-deficiency patients, this correlation was almost maintained (r = 0.481 and *p* = 0.059). The proportions of fast-twitch fibers negatively correlated with muscle SOD-2 protein levels among all COPD patients and in the iron-deficiency group (Figure 7B). Such a correlation was lost in the non-iron deficiency group of patients (Figure 7B). A significant increase in lipofuscin levels was detected in the vastus lateralis of the iron-deficiency COPD compared to the non-iron deficiency patients (Table 3, Figure 8). No significant differences were observed in the markers of muscle damage: total abnormal fraction, internal nuclei count, inflammatory cells, abnormal cells, necrotic cells, or apoptotic nuclei between the study groups.

## 4. Discussion

The study hypothesis has been confirmed in this study. Oxidative/nitrosative stress levels were greater in the muscle and blood compartments of COPD patients with iron deficiency. Importantly, levels of the powerful antioxidants SOD and TEAC were reduced in the vastus lateralis and the systemic compartment of the patients with iron depletion compared to those with normal iron content. The population of COPD patients recruited in the present study has been well characterized as determined by the lower levels observed in the markers of iron metabolism among patients with iron deficiency, whereas in patients with preserved iron content, the levels of those biomarkers were within normal ranges. The predominance of fast-twitch fibers within the vastus lateralis of the iron-depleted patients (almost 80%) compared to the control COPD patients was also a novel finding in the present investigation. All these results are discussed below.

The assessment of the systemic and muscle compartments from the same patients was a relevant approach in the study. Consistently, levels of protein tyrosine nitration as measured by the 3-nitrotyrosine marker were significantly greater in the lower limb muscles and systemic compartments of COPD patients with iron deficiency as compared to patients with normal iron content. These are relevant findings that point towards the existence of a crosstalk between the two compartments as also previously reported in a study [15] in which a similar approach was used. The confirmation of the crosstalk between these two specific compartments has clinical implications, since therapeutic strategies will be able to target the two compartments simultaneously.

Other relevant findings in the investigation were the significant decrease observed in the powerful antioxidants SOD and TEAC. SOD scavenges superoxide anion to produce hydrogen peroxide, a substrate for catalase enzyme activity. An excess of superoxide anion production leads to the generation of the powerful reactive nitrogen species (RNS) peroxynitrite resulting from its reaction with nitric oxide (NO). The reaction of peroxynitrite with aromatic amino acids (i.e., tyrosine) results in the formation of protein tyrosine nitration events in tissues [55,56]. Indeed, protein tyrosine nitration is the most relevant marker of nitrosative stress in vivo with a potential to inactivate enzymes or prevent phosphorylation of tyrosine kinase substrates [57]. The decrease in mitochondrial SOD observed in the vastus lateralis and blood compartment of the iron-deficiency patients may account for the increased levels of protein tyrosine nitration observed in the same compartments in these patients. These are very consistent findings from a biochemical standpoint. The mechanisms whereby iron depletion influences mitochondrial SOD content in both muscle and the systemic compartments warrant further attention in future investigations.

The biomarker TEAC measures the antioxidant capacity based on the compound Trolox (a water-soluble analog of vitamin E). Trolox equivalency is calculated on the basis of the ABTS (2,2′-azino-bis [3-ethylbenzothiazoline-6-sulfonic acid]) decolorization assay [58,59]. In the systemic compartment of the iron-depleted patients, TEAC levels were reduced compared to patients with normal iron content. As far as we are concerned, this is the first report in which TEAC levels were shown to be decreased in COPD patients with iron deficiency without anemia. Previous investigations also demonstrated a significant decrease in TEAC levels in the blood of patients with anemia of different etiology [60,61]. These findings are also in line with the decrease seen in the levels of mitochondrial SOD in the muscles of the patients with iron deficiency. Collectively, these results suggest that in stable COPD patients, iron deficiency is associated with a depleted antioxidant capacity in both muscle and systemic compartments.

The compound lipofuscin is a marker of unsaturated fatty acid oxidation, particularly of damaged membranes, mitochondria, and lysosomes. Additionally, lipofuscin may also contain sugars and metals such as iron, mercury, copper, and zinc [62]. Oxidized proteins may also be identified in the lipofuscin aggregates [63]. In general, lipofuscin inclusions are considered to be a marker of lipid peroxidation in tissues. They tend to accumulate in postmitotic cells as a marker of aging [64]. In the vastus lateralis of patients with severe COPD, lipofuscin aggregates were also significantly greater than in control subjects [65]. Consistently, in the current study, lipofuscin inclusions were significantly greater in muscles of the iron-deficient patients than in those of patients with normal iron content, suggesting that lipid peroxidation was probably more prominent among patients with iron deficiency. Whether the lipofuscin aggregates may contain higher levels of iron in these patients needs to be explored in future investigations.

A switch from the slow- to fast-twitch muscle phenotype has been consistently demonstrated in vastus lateralis patients with COPD, particularly in those with a severe disease [51,66,67]. Several factors, such as reduced physical activity, deconditioning, and malnourishment, play a crucial role in the development of a less fatigue-resistant phenotype of the lower limb muscles in COPD patients and in other chronic diseases [68,69,70,71,72]. In the present investigation, the switch to a less resistant phenotype was significantly more pronounced in patients with iron deficiency than in patients with normal iron content. This finding was independent of the muscle function, as no differences were seen in quadriceps muscle function between the two study groups. Furthermore, a significant positive correlation was observed between muscle levels of mitochondrial SOD and the proportions of slow-twitch fibers in all the patients as a whole and in the iron-deficient group. Interestingly, inverse relationships were also seen between protein levels of SOD2 and those of the fast-twitch muscle fibers among all the COPD patients as a group and in those with iron depletion. These are all relevant findings that suggest that mitochondrial SOD expression follows a specific fiber type distribution in the lower limb muscles of COPD patients and iron may be involved in the underlying mechanism. In fact, a decrease in the proportions of oxidative fibers was also reported in mice with iron deficiency [73], suggesting that the element iron is deeply involved in the maintenance of an adequate proportion of oxidative fibers within the muscles.

## 5. Study Limitations

A potential limitation in the study relies on its descriptive nature, which is based on the design of a cross-sectional investigation. Treatment with iron for several months may partly revert the effects seen in the systemic and muscle compartments of the patients. This would have required the design of a longitudinal study in which patients should be evaluated at different time-points, with the difficulties at the time of obtaining the biological specimens. Despite these potential limitations, we believe that the current investigation opens a new avenue of research in COPD patients with iron depletion. In addition, the model used in the study has enabled us to analyze the systemic and muscle compartments in the same population of COPD patients.

## 6. Conclusions

In severe COPD, nitrosative and oxidative stress along with a reduction in the antioxidant capacity were demonstrated in the vastus lateralis and systemic compartments of patients with iron deficiency without anemia. The slow- to fast-twitch muscle fiber switch towards a less resistant phenotype was also significantly more prominent in the muscles of these patients, suggesting that iron content may be involved in this phenomenon. Iron deficiency is associated with a specific pattern of oxidative and nitrosative stress and reduced antioxidant capacity in severe COPD irrespective of muscle function of the lower limbs. In the clinics, parameters of iron metabolism and content should be routinely quantify given its implications in redox balance and exercise tolerance. These results should also be taken into consideration in the design of therapeutic strategies in severe COPD, including pulmonary rehabilitation programs.

## Figures and Tables

**Table 1:** General clinical characteristics of the study patients.

	COPD Patients		
	Non-Iron Deficiency	Iron Deficiency	*p*-Value
	N = 20	N = 20	
Anthropometry			
Age (years)	67.3 ± 8.0	65.9 ± 8.1	0.602
Males/Females	12/8	12/8	1.000
Body weight (Kg)	59.72 ± 11.33	63.97 ± 13.07	0.280
BMI (Kg/m^2^)	22.73 ± 3.64	24.08 ± 4.28	0.301
FFMI (Kg/m^2^)	15.65 ± 2.30	14.72 ± 2.15	0.202
Smoking history			
Smoking status (active/ex-smoker)	9/11	12/8	0.515
Packs-year	53.2 ± 33.4	44.9 ± 24.1	0.406
Lung Function			
FEV_1_ (L)	1.20 ± 0.37	1.23 ± 0.41	0.759
FEV_1_ (% predicted)	47.26 ± 13.50	43.42 ± 11.13	0.345
FVC (L)	2.82 ± 0.50	2.76 ± 0.76	0.753
FVC (% predicted)	85.05 ± 14.52	76.74 ± 12.24	0.064
FEV_1_/FVC	45.19 ± 11.73	46.62 ± 11.44	0.706
GOLD classification			
1, (%)	0	0	0.224
2, (%)	45	25	
3, (%)	45	60	
4, (%)	10	15	
A, (%)	55	45	0.699
B, (%)	35	45	
C, (%)	5	5	
D, (%)	5	5	
Iron status			
Hemoglobin (g/dL)	15.23 ± 1.46	14.99 ± 1.64	0.641
Hematocrit (%)	45.40 ± 4.81	44.97 ± 4.45	0.778
MCV (fL)	93.84 ± 3.56	91.61 ± 6.84	0.214
MCH (pg)	31.52 ± 1.39	30.53 ± 2.78	0.176
MCHC (g/dL)	33.59 ± 1.26	33.31 ± 1.01	0.447
Ferritin (ng/mL)	212.16 ± 60.77	68.52 ± 32.62	0.000
Transferrin saturation (%)	31.45 ± 8.36	24.78 ± 7.02	0.016
Transferrin (g/dL)	237.26 ± 31.65	259.11 ± 28.71	0.032
Soluble transferrin receptor (mg/L)	2.18 ± 0.49	2.99 ± 0.80	0.001
Serum iron (µg/dL)	106.54 ± 26.84	91.44 ± 27.35	0.099
Hepcidin (ng/mL)	411.30 ± 113.92	87.78 ± 68.25	0.000

Data are presented as mean ± SD. Abbreviations: COPD, chronic obstructive pulmonary disease; BMI, body mass index; FFMI, fat-free mass index; N, number of patients; FEV_1_, forced expiratory volume in one second; FVC, forced vital capacity; MCV, mean corpuscular (erythrocyte) volume; MCH, mean corpuscular hemoglobin; MCHC, mean corpuscular hemoglobin concentration.

**Table 2:** Exercise and muscle function assessment of the study patients.

	COPD Patients		
	Non-Iron Deficiency	Iron Deficiency	*p*-Value
	N = 20	N = 20	
Six-minute walk test			
Distance (m)	481.67 ± 59.45	435.12 ± 57.82	0.025
Distance (% predicted)	98.61 ± 17.21	84.47 ± 14.56	0.013
Upper limb muscle strength			
D-HGS (Kg)	26.50 ± 6.98	27.78 ± 8.16	0.617
D-HGS (% predicted)	89.91 ± 15.48	91.20 ± 19.02	0.887
ND-HGS (Kg)	24.16 ± 7.36	24.56 ± 8.89	0.825
ND-HGS (% predicted)	91.44 ± 22.32	87.68 ± 19.57	0.595
Lower limb muscle strength			
D-QMVC (Kg)	22.12 ± 6.74	22.58 ± 4.35	0.831
D-QMVC (% predicted)	62.23 ± 22.00	63.81 ± 11.46	0.784
ND-QMVC (Kg)	21.44 ± 5.85	22.00 ± 4.86	0.825
ND-QMVC (% predicted)	60.79 ± 22.07	62.03 ± 12.32	0.858

Data are presented as mean ± SD. Abbreviations: COPD, chronic obstructive pulmonary disease; D, dominant; ND, non-dominant; HGS, handgrip strength; QMVC, quadriceps maximum voluntary contraction.

**Table 3:** Structural characteristics of vastus lateralis of the study patients.

	COPD Patients		
	Non-Iron Deficiency	Iron Deficiency	*p*-Value
	N = 18	N = 16	
Muscle fiber type proportions			
Type I fibers (%)	27.36 ± 7.70	20.04 ± 7.84	0.008
Type II fibers (%)	66.52 ± 10.11	76.85 ± 8.97	0.003
Hybrid fibers (%)	6.12 ± 7.01	3.11 ± 2.83	0.109
Cross-sectional fiber type areas			
Type I fibers (µm^2^)	2661.72 ± 717.11	2837.05 ± 889.59	0.539
Type II fibers (µm^2^)	1886.94 ± 645.03	1970.29 ± 781.53	0.744
Hybrid fibers (µm^2^)	1973.64 ± 987.46	2179.66 ± 1161.20	0.639
Muscle structural abnormalities	N = 20	N = 20	
Total abnormal fraction (%)	1.38 ± 0.81	1.70 ± 0.76	0.215
Internal nuclei count (%)	0.90 ± 0.42	1.12 ± 0.74	0.237
Inflammatory cells (%)	0.08 ± 0.08	0.10 ± 0.08	0.538
Lipofuscin (%)	0.01 ± 0.03	0.07 ± 0.10	0.014
Abnormal cells (%)	0.11 ± 0.15	0.15 ± 0.16	0.321
Necrotic cells (%)	0.30 ± 0.61	0.23 ± 0.40	0.698
Apoptotic nuclei (%)	53.48 ± 7.70	57.43 ± 8.81	0.139

Data are presented as mean ± SD. Abbreviations: COPD, chronic obstructive pulmonary disease; CSA, cross-sectional fiber area.

## Data Availability

The datasets generated and analyzed during the current study are available from the corresponding author on reasonable request.

## Supplementary Materials

The following supporting information can be downloaded at: https://www.mdpi.com/article/10.3390/nu15061454/s1; Figure S1: Standard curve Elabscience® 3-NT(3-Nytrotyrosine) ELISA kit; Figure S2: Standard curve— Protein Carbonyl Content Assay Kit; Figure S3: Standard curve— OxiSelectTM MDA Adduct Competitive ELISA Kit; Figure S4: Standard curve—Superoxide Dismutase Assay Kit; Figure S5: Standard curve—Catalase Assay kit; Figure S6: Standard curve—The OxiSelectTM TEAC (TEAC Assay Kit (ABTS); Figure S7: Standard curve—The human Reduced Glutathione (GSH) ELISA Kit; Figure S8: Standard curve—The Human hepcidin (Hepc) ELISA kit; Figure S9: Intra-assay coefficients of variation.
