# Supplementary material for: Nitrosative and Oxidative Stress, Reduced Antioxidant Capacity, and Fiber Type Switch in Iron-Deficient COPD Patients: Analysis of Muscle and Systemic Compartments
Source: Nutrients. 2023 Mar 17;15(6):1454. doi: 10.3390/nu15061454 (PMC10053245; doi:10.3390/nu15061454)
Supplement: Supplementary file 1 [file nutrients-15-01454-s001.zip › nutrients-2251995-supplementary.pdf]

Figure S1. Standard curve - Elabscience® 3-NT(3-Nytrotyrosine) ELISA kit

Standard curve	
ng/mL	OD
100.00	0.12
50.00	0.16
25.00	0.23
12.50	0.31
6.25	0.46
3.13	0.61
1.56	0.71
0.00	0.83

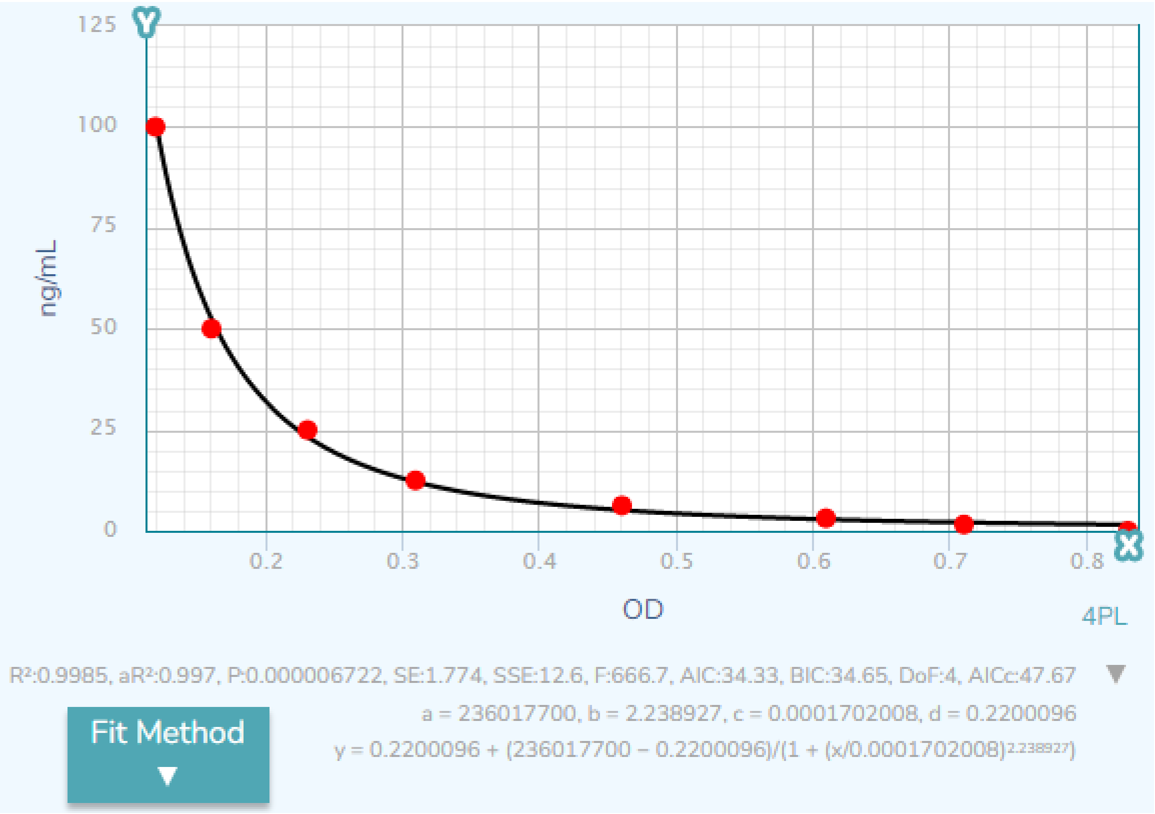

Figure S2. Standard curve - Protein Carbonyl Content Assay Kit

Standard curve	
**ng/mL**	**OD**
2000	0.93
1500	0.73
1000	0.53
750	0.41
500	0.30
250	0.17
125	0.09
25	0.01
0	0.00

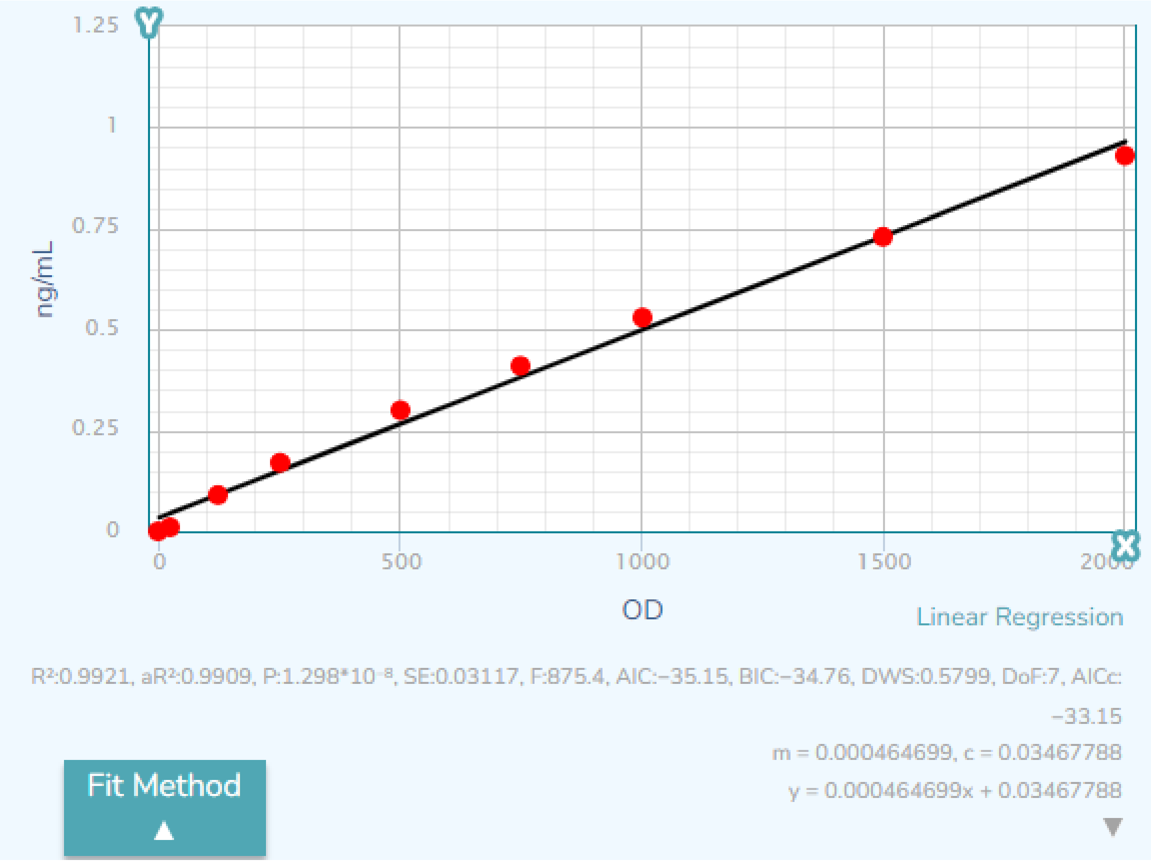

Figure S3. Standard curve - OxiSelect™ MDA Adduct Competitive ELISA Kit

Standard curve	
ng/mL	OD
1500	0.05
188	0.09
94	0.12
47	0.16
24	0.19
12	0.20
6	0.22
0	0.25

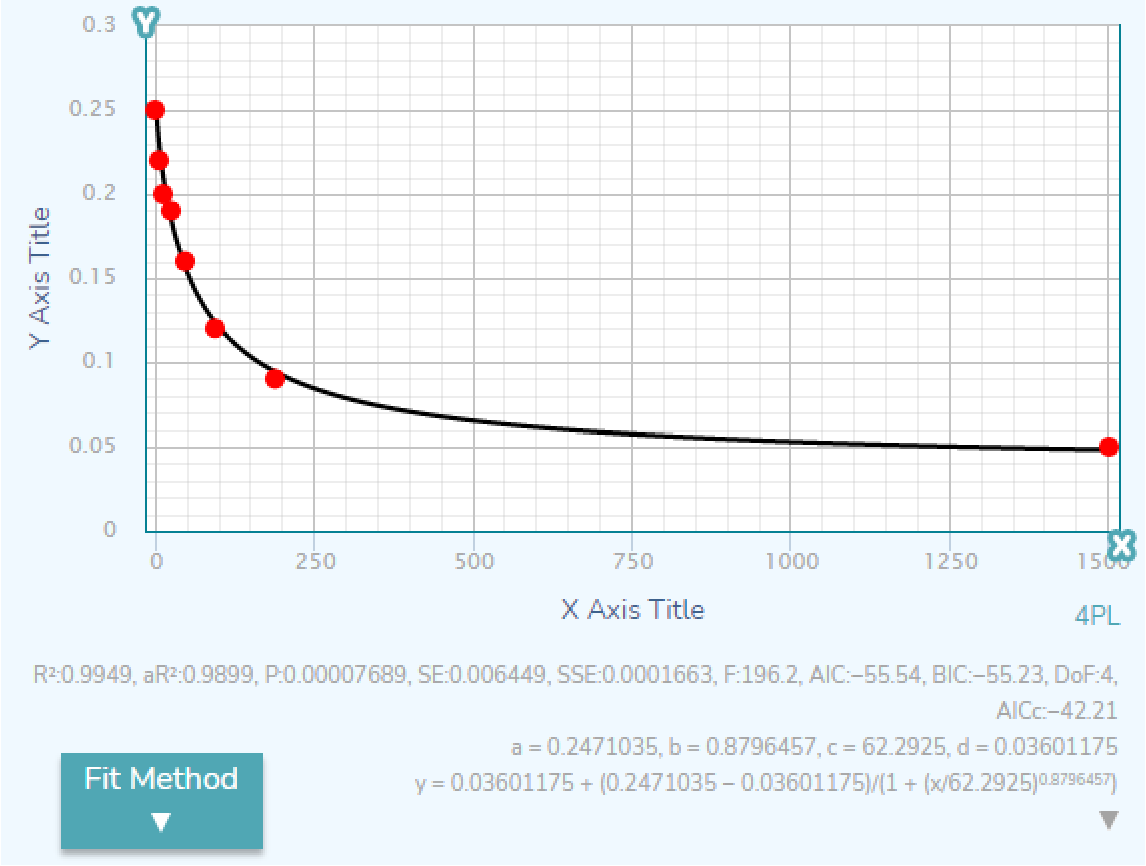

Figure S4. Standard curve - Superoxide Dismutase Assay Kit

Standard curve	
U/mL	OD
0	0.35
0.005	0.31
0.01	0.28
0.015	0.24
0.02	0.20
0.025	0.18
0.03	0.16
0.04	0.10

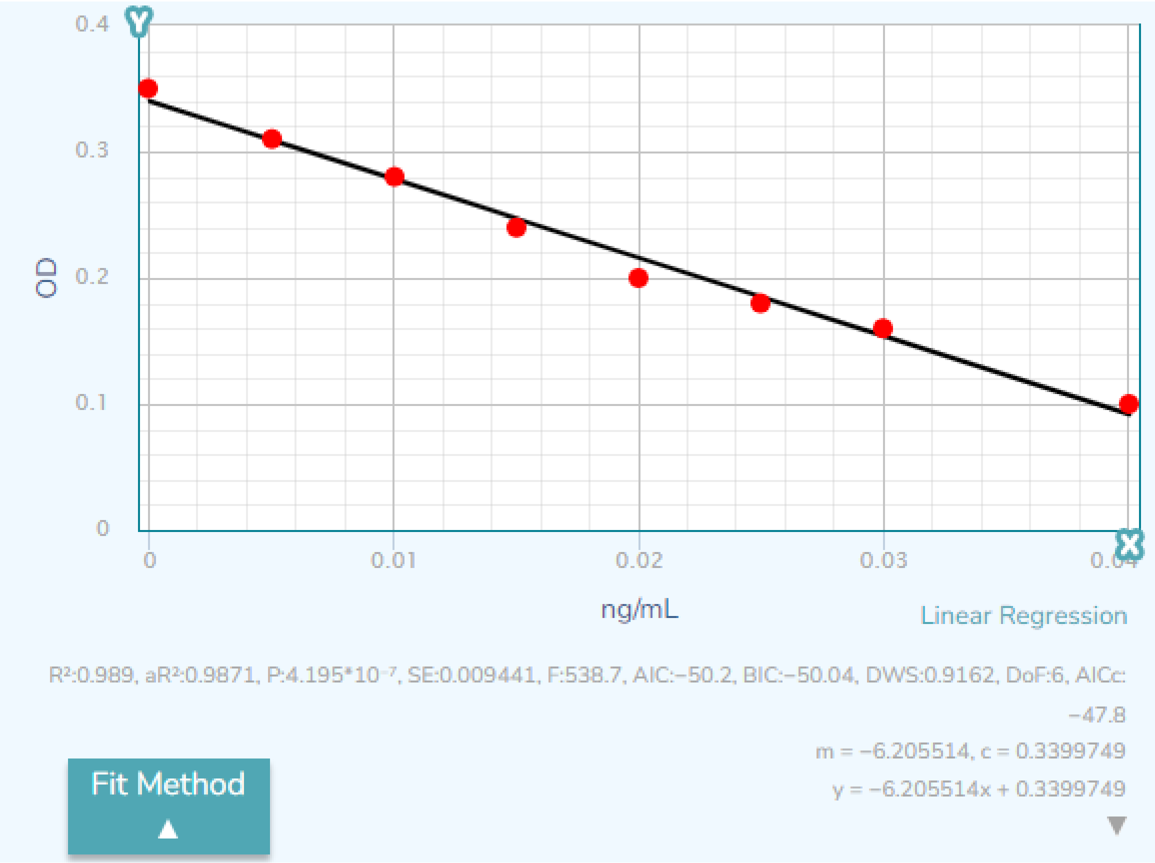

Figure S5. Standard curve - Catalase Assay kit

Standard curve	
U/mL	OD
0.00	0.00
5.00	0.07
10.00	0.09
15.00	0.15
20.00	0.19
30.00	0.28
45.00	0.40
60.00	0.49
75.00	0.68
100.00	0.86

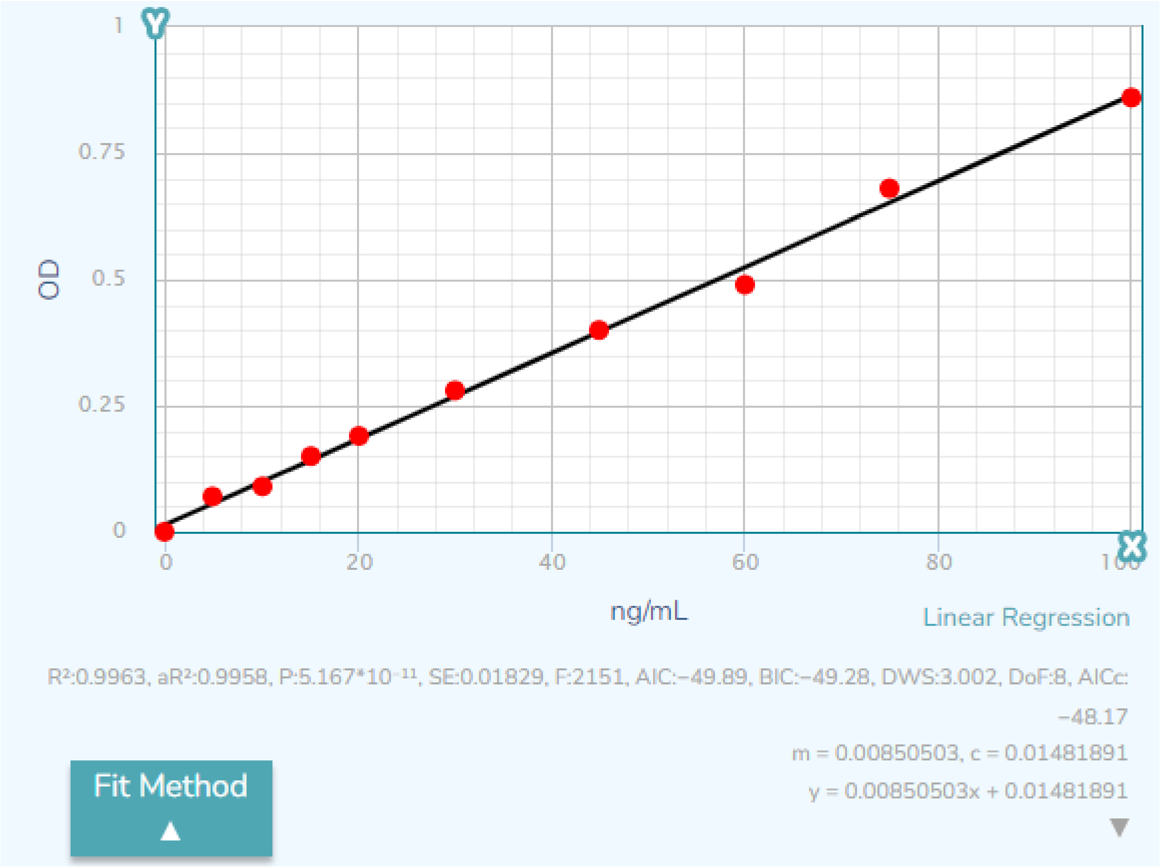

Figure S6. Standard curve - The OxiSelectTM TEAC (TEAC Assay Kit (ABTS)

Standard curve	
(TE) µmol/g	OD
150.00	0.528
75.00	0.826
37.50	0.965
18.80	1.084
9.40	1.138
4.70	1.158
0.00	1.171

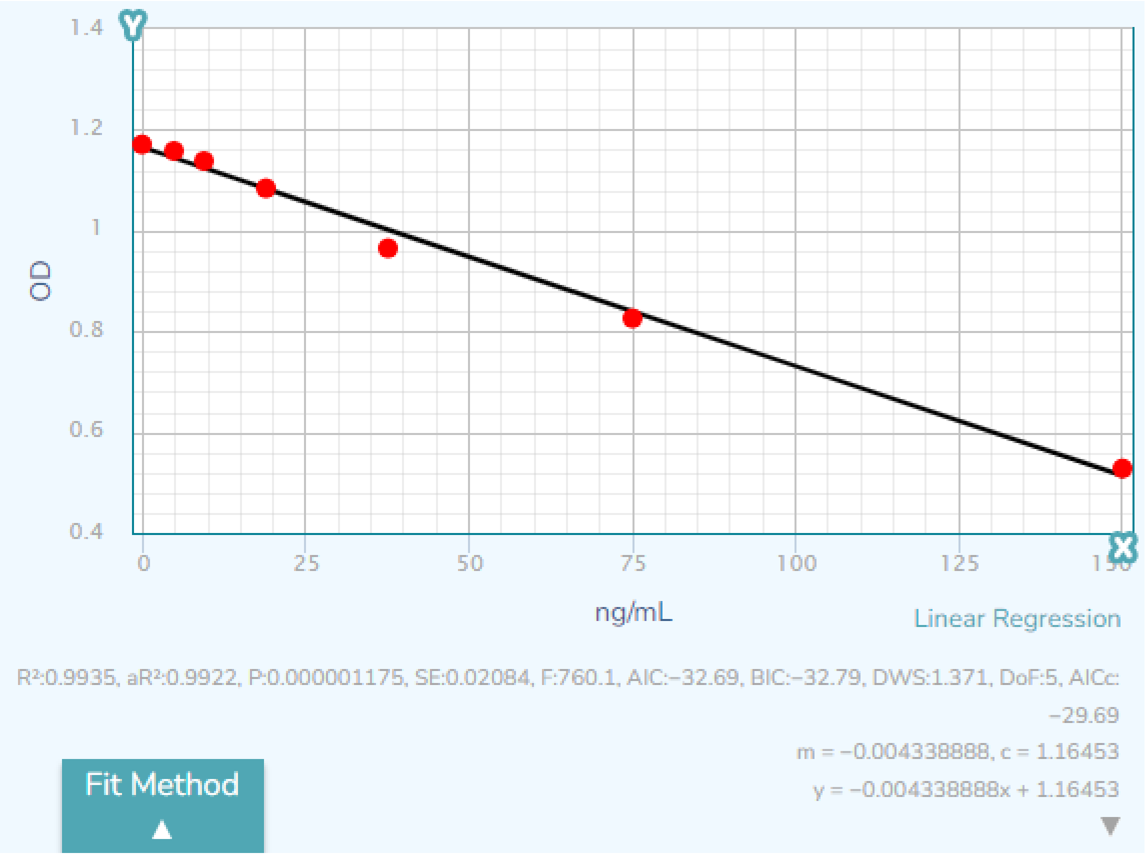

Figure S7. Standard curve - The human Reduced Glutathione (GSH) ELISA Kit

Standard curve	
ng/mL	OD
0	0.93
1.56	0.67
3.125	0.38
6.25	0.22
12.5	0.15
25	0.10
37.5	0.09
50	0.06

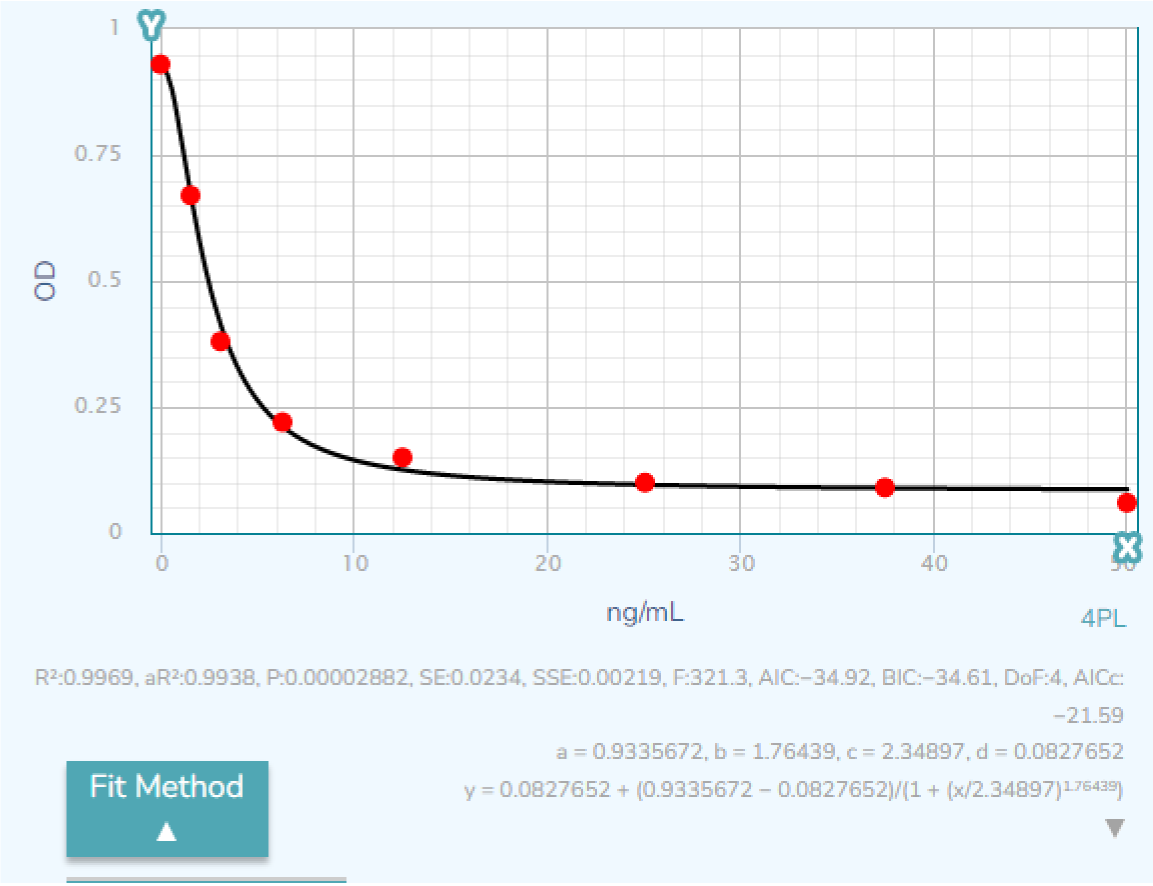

Figure S8. Standard curve - the Human hepcidin (Hepc) ELISA kit

Standard curve	
ng/mL	OD
0	0
12.5	0.07075
25	0.1149
37.5	0.16755
50	0.22685
87.5	0.3685
100	0.5512
200	0.7719
300	1.3383
400	1.3994

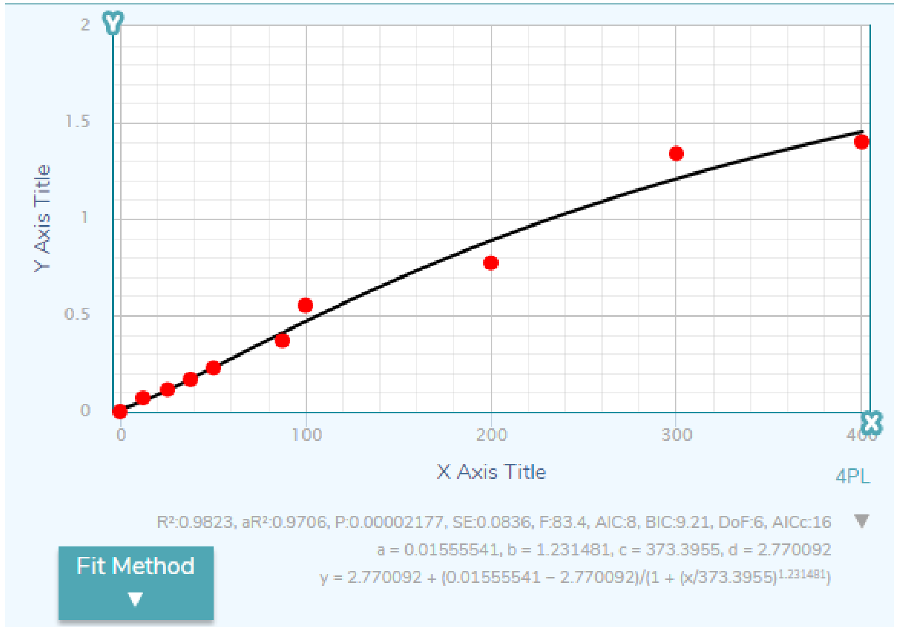

Figure S9. Intra-assay CV

Intra-assay coefficients of variation								
Sample ID	TEAC	GSH	Catalase	SOD	3-NT	Protein carbonylation	MDA protein adducts	Hepcidin
1	1.83	3.02	4.00	0.48	0.86	0.80	2.16	5.18
2	1.44	0.61	4.74	3.47	9.81	1.07	3.91	9.18
3	1.02	2.23	3.85	4.85	1.19	2.95	0.68	1.63
4	0.86	3.98	2.68	1.53	3.76	3.59	1.30	6.58
5	2.57	2.18	3.92	4.26	4.56	1.73	2.54	4.75
6	3.08	7.78	1.74	4.58	1.82	4.02	1.67	4.34
7	1.89	3.32	3.91	3.04	7.70	0.81	2.42	2.31
8	0.82	2.01	3.86	0.22	5.53	5.57	0.40	1.20
9	0.08	1.38	1.71	1.33	6.78	2.90	1.07	6.41
10	1.85	3.28	0.47	0.38	4.03	0.68	3.55	1.59
11	0.88	1.61	7.97	2.29	6.99	5.80	4.41	2.41
12	3.13	3.99	3.29	1.96	1.51	0.25	5.29	0.11
13	4.35	0.06	5.78	0.71	1.27	4.74	2.04	4.38
14	1.92	0.18	8.52	4.64	4.65	2.31	2.13	2.62
15	2.11	0.00	2.14	0.99	0.86	2.03	4.88	5.04
16	0.65	1.94	2.86	2.18	4.65	4.94	0.24	3.83
17	1.14	1.55	0.96	4.22	2.06	3.92	1.89	1.99
18	2.24	0.09	8.83	8.92	4.51	0.79	7.18	4.96
19	1.44	1.45	5.35	6.37	2.60	3.92	4.33	3.33
20	2.99	1.05	7.17	0.17	2.21	0.98	1.61	3.34
21	1.85	1.44	4.97	0.12	0.27	1.18	6.64	6.60
22	2.89	0.07	2.83	3.58	2.92	0.43	1.68	0.84
23	3.10	0.76	4.05	3.31	1.85	1.64	0.13	5.20
24	0.73	0.51	3.63	2.84	7.03	1.40	2.33	4.03
25	3.64	1.93	8.49	6.03	0.61	0.24	1.30	5.57
26	0.07	1.90	3.54	2.37	4.76	1.71	3.34	4.64
27	3.13	0.89	8.63	8.45	3.03	3.10	0.66	3.08
28	2.80	4.63	7.06	3.90	1.89	0.00	2.33	10.98
29	0.83	1.06	9.25	1.25	1.94	1.82	0.70	3.20
30	0.78	0.50	3.57	0.94	6.82	1.09	1.30	4.53
31	0.75	1.84	4.64	0.49	3.93	6.31	2.70	4.88
32	3.75	3.64	6.19	4.54	2.31	0.83	3.01	4.35
33	4.22	3.81	7.03	4.93	0.87	0.00	9.74	2.72
34	1.31	1.53	1.65	2.07	7.16	3.42	3.81	1.24
35	2.55	2.79	0.42	3.32	5.23	0.85	1.00	6.28
36	2.04	4.02	3.45	5.31	2.75	1.41	8.36	0.15
37	5.28	1.57	7.06	5.40	0.51	0.90	4.99	9.40
38	2.24	1.62	5.19	8.43	0.56	0.76	1.28	9.49
39	3.05	1.57	4.31	3.46	1.67	6.43	2.10	6.52
40	1.69	4.08	2.54	1.50	3.62	1.24	7.25	8.86
